# Review on Current Status of Echinocandins Use

**DOI:** 10.3390/antibiotics9050227

**Published:** 2020-05-02

**Authors:** Martyna Mroczyńska, Anna Brillowska-Dąbrowska

**Affiliations:** Department of Molecular Biotechnology and Microbiology, Gdańsk University of Technology, Narutowicza 11/12, 80-233 Gdańsk, Poland; martyna.mroczynska@pg.edu.pl

**Keywords:** antimycotic, fungal infections, echinocandin resistance

## Abstract

Fungal infections are rising all over the world every year. There are only five medical compound classes for treatment: triazoles, echinocandins, polyenes, flucytosine and allylamine. Currently, echinocandins are the most important compounds, because of their wide activity spectrum and much lower sides effects that may occur during therapy with other drugs. Echinocandins are secondary metabolites of fungi, which can inhibit the biosynthesis of β-(1,3)-D-glucan. These compounds have fungicidal and fungistatic activity depending on different genera of fungi, against which they are used. Echinocandin resistance is rare—the major cause of resistance is mutations in the gene encoding the β-(1,3)-D-glucan synthase enzyme. In this review of the literature we have summarized the characteristics of echinocandins, the mechanism of their antifungal activity with pharmacokinetics and pharmacodynamics, and the resistance issue.

## 1. Introduction

Fungal infections contribute to the deaths of over 1.5 million people around the world each year. About 90% of fungal infection-related deaths are caused by species belonging to four fungal genera: *Cryptococcus*, *Candida*, *Aspergillus* or *Pneumocystis*. Immunosuppressive therapies associated with organ transplants, and diseases, such as AIDS or cancer, have contributed to the growth of fungal infections over the years [1]. Epidemiological studies in the United States have shown that fungi from *Candida* spp. are the fourth most common pathogens acquired in hospitals that cause bloodstream infections. In terms of incidence, *Candida* spp. infection is estimated to occur in 6 to 13.3 cases per 100,000 inhabitants [2]. There are five classes of antifungal agents: triazoles, echinocandins, polyenes, flucytosine and allylamine. The mechanism of triazole action involves inhibiting synthesis of plasma membrane ergosterol [3]. Fluconazole, itraconazole, voriconazole and posaconazole are among the triazole antibiotics. Echinocandin drugs inhibit the glucan synthase enzyme, resulting in the inhibition of glucan biosynthesis, which is part of the fungal cell wall. Examples of echinocandin antifungal medications include caspofungin, micafungin and anidulafungin [3]. Amphotericin B, a member of the polyene class of antifungals, binds to ergosterol and causes changes in cell membrane permeability. Flucytosine—a pyrimidine analogue—converts to 5-fluorouracil, which becomes integrated during RNA synthesis causing early chain termination and blocking the process of DNA synthesis [4]. Allylamine (e.g., terbinafine) inhibits the action of squalene epoxidase, an enzyme important for the conversion of squalene to squalene-2,3-epoxid, which is involved in the ergosterol synthesis pathway. Moreover, the high level of squalene is toxic for the cell and can cause pH imbalance [5].

The purpose of this review is to gather the most important information on echinocandins with respect to their application in fungal infections treatment. 

## 2. Echinocandins

Echinocandins are secondary metabolites of fungi that contain a core composed of a cyclic hexapeptide and lipid residues responsible for their antifungal activity. In the 1970s, two compounds, echinocandin B and aculeacin A, were identified as antifungal agents. Cilofungin, a synthetic version of echinocandin B, was withdrawn from the second phase of a clinical trial due to high levels of toxicity [4]. Anidulafungin was discovered in 1970, and the precursors of caspofungin and micafungin were described in 1989 and 1990, respectively [6,7]. Echinocandins are recommended as first-line treatments in patients suffering from invasive *Candida* infection, particularly in hemodynamically unstable patients after prior treatment with triazoles [8,9]. 

### 2.1. Semi-Synthetic Echinocandin Derivatives

#### 2.1.1. Caspofungin

**Figure 1:**
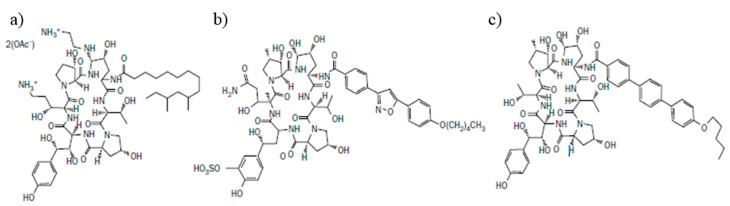
The chemical structure of (**a**) caspofungin, (**b**) micafungin, (**c**) anidulafungin.

Caspofungin is a 1-[(4R, 5S)-5-[(2-aminoethyl) amino]15-N2-(10,12-dimethyl-1-oxotetradecyl)-4-hydroxy-L-ornithine]-5-[(3R)-3-hydroxy-L-ornithine] pneumocandin B0 diacetate. Figure 1a presents the chemical structure of caspofungin. The Food and Drug Administration (FDA) approved caspofungin in January 2001 as a drug used to prevent fungal infections in adult patients. In July 2008, it was approved for use in children over 3 months old [7]. This compound is a derivative of a naturally occurring hexapeptide in *Glarea lozoyensis*, modified by the addition of the N-acylated fatty acid chain as a side residue [7]. Currently, caspofungin is used in neutropenic patients who have high fever and are suspected to have fungal infection. It can be used to treat esophageal candidiasis, peritonitis, intra-abdominal abscess, and cavity infections caused by *Candida* [10]. Moreover, caspofungin is used as an alternative medicine when standard triazole therapy against *Aspergillus* spp. infections is not effective [8].

#### 2.1.2. Micafungin

Cleavage and the addition of an N-acylated side chain to a naturally occurring hexapeptide derived from *Coleophoma empetri* leads to the formation of a compound called micafungin (Figure 1b). Micafungin sodium is a 1-[(4R,5R)-4,5-dihydroxy-N2-[4-[5-[4-(pentyloxy)phenyl]-3–24 isoxazolyl]benzoyl]-L-ornithine]-4-[(4S)-4-hydroxy-4-[4-hydroxy-3-(sulfooxy)phenyl]-25 L-threonine], monosodium salt. This compound was approved by the FDA in March 2005 as a drug with antifungal activity [11]. Micafungin is used to treat patients suffering from esophageal candidiasis, and is also used as a prophylactic treatment against *Candida* infections in patients undergoing hematopoietic stem cell transplantation during neutropenia [8]. Micafungin was approved for pediatric patients aged 4 months and older suffering from *Candida* infections in 2013. Moreover, this compound was recently approved for treatment of invasive candidiasis in patients aged under 4 months [12]. 

#### 2.1.3. Anidulafungin

Anidulafungin (Figure 1c) is a derivative of echinocandin B, which is the fermentation product of *Aspergillus nidulans*. It is a 1-[(4R, 5R)-4,5-dihydroxy-N2-[[4″-(pentyloxy) [1,1′:4′, 1″-terphenyl]-4-yl]carbonyl]L-ornithine] echinocandin B. As an antifungal compound for the treatment of esophageal candidiasis, candidemia and deep-tissue candidiasis, anidulafungin was approved in February 2006 by the FDA [7]. The safety and the efficacy of anidulafungin in children and patients under 18 years have not been established. However, the first prospective study of safety and efficacy of anidulafungin in pediatric patients aged 1 month to 2 years has been recently published [13]. According to this research, no deaths were reported due to anidulafungin usage, moreover the results were comparable to the reported pharmacokinetic parameters in adults. Another study of anidulafungin application in the treatment of invasive candidiasis in children aged 2 to <18 years also reported this compound as effective and safe in pediatric patients [14]. 

## 3. The Mechanism of Action

**Figure 2:**
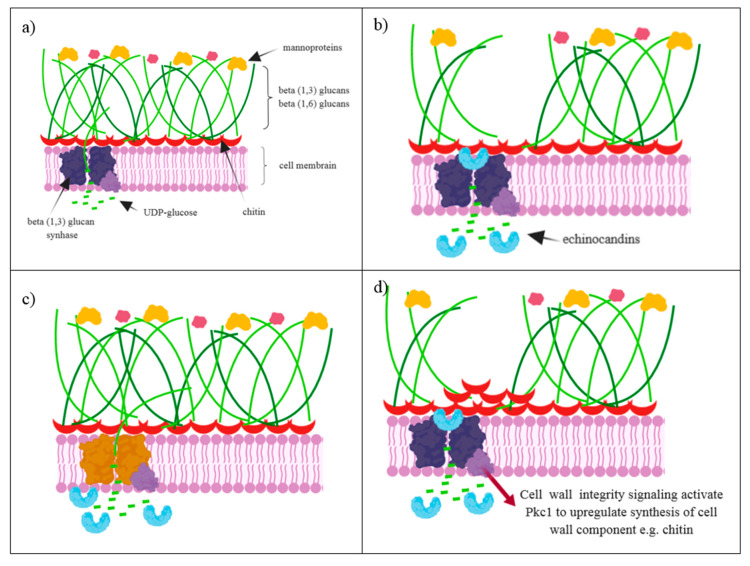
(**a**) The structure of a fungal cell wall; (**b**) Echinocandins act as noncompetitive inhibitors of β-(1,3)-D-glucan synthase. Inability of the microorganism to biosynthesize β-(1,3)-D-glucans leads to osmotic instability and cell death; (**c**) Nucleotides substitutions in the gene encoding glucan synthase contribute to the lack of echinocandin interactions with the enzyme; (**d**) Another mechanism of echinocandin resistance is to cell wall integrity, which is activated by RHO1 protein. The PKC, MAPK and calcineurin signaling pathways coordinate the regulation of the expression of chitin synthase gene and chitin synthesis.

The fungal cell wall consists of β- (1,3) -D-glucan polysaccharides, β- (1,4) -D-glucan, β-(1,6)-D-glucan, chitin, mannan, galactomannan, α -glucans and various glycoproteins (Figure 2a). In the structure of mammalian cells, the above-mentioned elements were not observed, and thus the components of the fungal cell wall are a good target for antimycotics [15,16]. 

The molecular target of echinocandins is UDP-glucose (1,3)-D-glucan-β-(3)-D-glucosyltransferase (commonly referred to as β-(1,3)-D-glucan synthase). This enzyme is responsible for the synthesis of β-(1,3-D)-glucan (homopolymer of β-D-glucopyranose, bonded by β-(1,3)-glycosidic bond), an important component of the cell walls of many fungi (Figure 2b) [17]. Together with chitin, these components confer the shape and integrity of the cell wall [18]. The β-(1,3)-D-glucan synthase is an integral membrane protein that catalyzes the reaction of the formation of a glucan polymer from UDP-glucose molecules.

In *Saccharomyces cerevisiae*, *FKS1* and *FKS2* genes encode highly homologous (87% identity) catalytic subunits of glucan synthase [19]. The glucan synthase enzyme is a multienzyme complex, consisting of an integral membrane protein catalytic subunit (FKS) with regulatory subunit RHO1 protein [20]. This complex is also described in *Candida albicans*, nevertheless FKS2 protein homolog is not transcribed in growing cells [4]. The third homolog, *FKS3*, is expressed at a low level and probably does not affect the level of glucan biosynthesis [3,21]. In addition, *FKS1* gene transcription is regulated by the cell cycle and is associated with the reconstruction of the fungal cell wall, whereas the expression of the *FKS2* gene is dependent on calcineurin [6,7]. That key regulatory protein seems to be the expression product of the *RHO1* gene, which interacts with both FKS proteins and protein kinase C (PKC). RHO1 protein can regulate the cascade of mitogen-activated protein kinases (MAPK), as well as the cytoskeletal actin synthesis pathway in yeast. Due to the high number of interactions with various proteins, it is suspected that RHO1 protein is an activator of glucan synthesis [6].

### 3.1. Antifungal Activity of Echinocandins

The implementation of effective in vivo therapy is based on the earlier in vitro studies of the susceptibility of the pathogen to antibiotics. In order to determine the level of antibiotic sensitivity, Minimal Inhibitory Concentration (MIC) or Minimal Effective Concentration (MEC) values were tested using the microdilution method, according to the current European Committee for Antimicrobial Susceptibility Testing (EUCAST) guidelines [22]. MIC is the minimum concentration of an antifungal agent that inhibits mycelial growth. MEC is defined as the lowest concentration of a compound that causes the growth of aberrant, short, hyphal segments (comparing to the growth control), e.g., *Aspergillus* spp. [7]. The results of multicenter sensitivity studies, as well as knowledge of resistance mechanism action, are considered in determining clinical breakpoints. The new guidelines were published by EUCAST in February 2020 [23]. In Table 1 there are presented breakpoint values for anidulafungin (AND), caspofungin (CAS) and micafungin (MCF) against *Candida* spp., established by both EUCAST and CLSI (Clinical & Laboratory Standards Institute). The new EUCAST guidelines underline that in cases where the *C. albicans*’ isolates of micafungin’s MIC value equals 0.03 mg/L, anidulafungin MIC values should be considered to classify isolates as MCF resistant or susceptible. Anidulafungin sensitive isolates (≤0.016 mg/L) should also be identified as sensitive to micafungin, even if MIC values of MCF equal to 0.03 mg/L. However, in cases of *C. albicans* resistant to anidulafungin, resistance to MCF can be concluded only in cases where the presence of mutations in the *FKS* gene is confirmed. Caspofungin breakpoints have not yet been established because of significant inter-laboratory variation of MIC values [24].

Echinocandins show fungicidal activity against both *C. albicans* and a large group of non-*albicans* species (Table 2), including species such as *C. glabrata* and *C. krusei*, which are intrinsically resistance to triazoles, and *C. lusitaniae* resistance to amphotericin B [3,7]. These drugs are also effective on yeasts, which are able to produce a biofilm [3]. In some *Candida* species, echinocandins destabilize the cell wall integrity and reduce its stiffness. As a consequence, cell lysis occurs due to low resistance to osmotic pressure [18]. Antibiotics from the echinocandin group are used in the first-line treatment in patients with invasive candidiasis. Epidemiological studies performed in the United States report that more than 60% of patients with candidiasis are treated with this drug [25]. Echinocandins are also recommended as the first-line therapy against multidrug resistant *C. auris* [26]. However, caspofungin is not active against *C. auris* biofilm [27]. Echinocandins are not used against renal tract or urinary tract *C. auris* infection, due to the failure to achieve therapeutic concentrations of the compound in urine. 

In cases of *Aspergilli*, echinocandins have fungistatic activity and reduce invasion via damage of hyphae and branching (Table 2). Determination of the MIC value for *Aspergillus* spp. can be challenging, therefore the determination of the MEC value is used in echinocandin susceptibility testing [7,28]. Anidulafungin exhibits the highest activity against *Aspergillus* spp. as compared to caspofungin and micafungin [28]. Surprisingly, *A. lentulus*, which shows a reduced susceptibility to most antifungal drugs, is sensitive to micafungin and anidulafungin; nevertheless, *A. lentulus* is less affected by caspofungin. During the analysis of *A. lentulus FKS1* gene, no polymorphism was found within the "hot spot" regions, hence it is suspected that the isolates use a resistance mechanism to antibiotics that has not been recognized yet. What has been proven is that these strains are capable of overproducing the glucans, limiting the effectiveness of the antibiotic [29,30]. Other studies conducted in 2015 showed that exposure of *A. fumigatus* to caspofungin caused an increased level of expression of the glucan synthase, and thus increased hyperbranched and chitin-rich hyphae. These hyphae are characterized by longer survival, acting as a biomass reservoir, which contributes to the growth of mycelium after antibiotics treatment [31]. 

Echinocandins used without additional antifungal compounds are not effective in the treatment against fungi of the genus *Mucorales*, *Fusarium*, *Rizpous*, *Scedosporium* and *Trichosporon* (Table 2) due to decreased amount of β-(1,3)-D-glucan, as mainly β-(1,6)-D-glucan is present in the cell walls of these fungi [7,17]. *Cryptococcus neoformans* also does not show high sensitivity to echinocandins in vitro. The cell wall of this microorganism consists mainly of α-(1,3)- or α- (1,6)-D-glucan, which is probably responsible for the decreased sensitivity to echinocandins [16]. It is also suspected that melanin may play an important role in protecting *C. neoformans* cells against the effects of antibiotics [30,32,33]. Additionally, other factors, such as modification or degradation of antibiotics, may also affect the antifungal activity, however, this theory has not been proven [8]. Echinocandins have variable activity against dimorphic fungi, depending on the form of growth. Echinocandins are active on the hyphal form of *Histoplasma*, but against the yeast form are less effective [17]. Research showed that in the yeast form of *Blastomyces* there is a low level of β-(1,3)-glucan [34]. It seems that this is the reason for the variable susceptibility of *Histoplasma* and *Blastomyces* isolates to echinocandin.

In vitro studies showed variable activity of echinocandin against *Alternaria* spp. and *Acremonium strictum*. However, evidence suggests that echinocandin should not be used against this fungi [35]. 

The recent report of *Coccidioides* susceptibility testing suggested that these fungi are susceptible to micafungin and anidulafungin, and less susceptible to caspofungin [36]. The previous research indicated the good response to caspofungin in mice infected with *C. immitis* [37]. However, coccidioidomycosis is a long-treatment disease, and the therapy takes several months. Due to intravenous delivery and high cost, caspofungin is not commonly used in the treatment of *C. immitis* infections. 

The in vitro examination of echinocandins can sometimes produce unexpected results. Stevens et al. observed that the *C. albicans* isolate grows in the presence of very high concentrations of caspofungin, which significantly exceeded the determined MIC values [46]. A similar effect can be observed for other strains of the genus *Candida*, such as *C. parapsilosis*, *C. tropicalis* and *C. krusei* [30,47]. These strains show usual sensitivity to a certain concentration, determined by means of the MIC value, while at a concentration exceeding the MIC value a “paradoxical effect” of mycelium growth occurs. Notably, this effect is very rare among *C. glabrata* strains [30]. The studies of Stevens et al. have shown that this effect is not related to the modification of the glucan synthase enzyme, i.e., mutations within *FKS* genes, nor increased biosynthesis of this enzyme [48]. It is suspected that reduced sensitivity to high concentrations of echinocandins may be connected with the adaptation of some fungi to stress. Another explanation may be the overproduction of chitin, which could supplement the deficiency of glucan in the cell wall [49,50]. This paradoxical effect is also observed with *A. fumigatus* fungi during exposure to high concentrations of caspofungin (> 1 mg/L). This effect does not occur with the other two echinocandins. Jurvadi et al. in 2015 showed that *A. fumigatus* activates the calmodulin and calcineurin pathway in response to high concentrations of caspofungin [51]. To activate these pathways, a high Ca^2+^ concentration is required in the cytosol, which was observed in *A. fumigatus* cells during exposure to caspofungin. Such high Ca^2+^ concentrations, capable of activating the calcineurin pathway, were not observed when cells were treated by micafungin, hence an absence of paradoxical growth was observed [51]. This effect also occurs during the clinical treatment of patients suffering from invasive lung aspergillosis, in which an increase in fungal antigens was observed during treatment with caspofungin [52].

Studies also show that echinocandins can be used in antifungal prophylaxis in patients after bone marrow or liver transplantation. The use of micafungin in adults and children with neutropenia after autologous or allogeneic transplants is 80% effective, while for fluconazole the efficacy reaches 74%. However, only 7.3% of transplant recipients who received treatment with caspofungin were infected with mold fungi [7]. In the case of liver transplants, with no preventive treatment, invasive infections in 20% of patients were found [53]. The prophylactic use of caspofungin (50 mg daily) for at least 21 days can drastically reduce the disease incidence. For example, according to a study by Fortún et al. concerning 71 patients with this type of prophylaxis for 19–41 days, only 2.8% of patients were infected [53]. Caspofungin as well as fluconazole have similar effects in the prevention of invasive fungal infections in high-risk patients after liver transplantation. However, according to many studies, caspofungin is safer for dialysis patients and has a high probability of invasive fungal infections [53]. The use of echinocandins among children is justified in the presence of *Candida* spp. infections (abdominal abscess, peritonitis, pleurisy and oesophagitis), as well as during therapy against *Aspergillus* spp. resistant to voriconazole and polyenes [54].

The use of echinocandins combined with other therapies is a promising avenue of research. Initially, the results of in vitro and animal models gave promising results [55,56]. For example, anidulafungin and voriconazole led to a reduction in mortality compared to monotherapy in some animal models [57]. Clinical studies have also confirmed a reduction in mortality subgroups of patients with invasive aspergillosis when using combined therapy [58]. The results of retrospective studies show that mixed therapy with voriconazole and caspofungin gave better results than the use of only voriconazole, which preceded treatment with various echinocandins or amphotericin B [59]. However, not all in vitro studies gave the same positive results. According to a study by Kirkpatrick et al., voriconazole treatment of patients gives better results than the combination therapy with caspofungin [60]. A 2015 study showed that the concomitant use of voriconazole and caspofungin does not improve the efficacy of treatment against *A. fumigatus* infections [61]. In order to be able to use combined therapy, many questions about the safety and efficacy of this type of therapy should be answered [62].

### 3.2. Pharmacokinetics and Pharmacodynamics

Echinocandins are distinguished by high molecular weight, a factor that contributes to problems with the absorption of the drug during oral dosing, which is why echinocandins have been approved as intravenous drugs [18]. The pharmacokinetics of echinocandins after intravenous administration have been well described. Perlin and Hope have shown that about 92% of the drug after a single dose is delivered to the tissues within 48 h. During the first 30 h a small amount of compound is excreted or biotransformed [62]. Caspofungin is metabolized by hydrolysis and N-acylation [30]. It also undergoes spontaneous chemical degradation, resulting in the formation of a microbiologically inactive compound with an open ring [63]. Anidulafungin is delivered to tissues in a short time, but it has a longer lifetime in the body. Its concentration in tissues, such as the liver, spleen, lungs or kidneys, is 10-fold higher than in plasma. The half-life of one dose of anidulafungin given once a day is between 1 and 2 days [62]. Elimination of the antibiotic in the organisms proceeds through chemical degradation. The presence of only 1% of anidulafungin in the urine, and as much as 30% of this antibiotic in the feces, was established [15,62]. Due to the lack of degradation of this compound by the liver and urinary excretion, this compound is safe in therapy for people with renal or hepatic insufficiency [64]. Distribution of micafungin to tissues occurs in a very short time, even less than five minutes, with the highest concentration of this drug detected in the lungs and kidneys. The concentration of micafungin in plasma decreases exponentially over time. The half-life of echinocandin is between 4 and 6 h [62]. Micafungin degradation occurs in two stages: (1) arylsulfatases are used and a catechol derivative is formed, and (2) the catechol-O-methyltransferase enzyme is responsible for the formation of the methoxy derivative [65].

Recent studies have allowed partial determination of plasma drug concentration, which will be optimal for the effective treatment of infections with the use of echinocandins. Fungicidal action against *Candida* spp. is observed in vitro over a wide range of concentrations. Studies in mice suffering from systemic candidiasis have shown that the most effective concentration of antibiotic is correlated with the ratio of maximum antibiotic concentration (C_max_)/MIC, or the area under the curve that determines plasma antibiotic concentration up to MIC. In the case of the *Aspergillus* genus, the pharmacodynamic parameters are not clearly defined. The best fungal action of echinocandins is associated with the concentration of the drug administered to the patient, determined by the ratio of C_max_/MEC [7].

### 3.3. Side Effects of Echinocandins

The side effects of treatment with echinocandins are comparable to side effects observed using fluconazole, and definitely less significant than in the case of amphotericin B [6,64]. Side effects which can lead to decision on discontinuation of the drug occur less often than in other antifungal drugs [7]. The most common complications directly associated with the infusion of the drug may include facial flushing, swelling, rash, pruritus, thrombophlebitis, hypotension and fever. These symptoms can be observed with all three echinocandins, varying in patients [7,64]. For example, fever is a frequent side effect for up to 30% of patients treated with caspofungin, while extremely rare (approximately 1% of patients) during the therapy with micafungin [6]. In order to reduce the adverse effects, the speed of drug application can be reduced [7,64]. Gastrointestinal problems, such as nausea, vomiting and diarrhea, are common side effects that occur in less than 7% of patients, and 3–25% of patients treated with caspofungin have phlebitis, while less than 2% of patients experience this condition when using anidulafungin and micafungin [17]. Anemia, leukopenia, neutropenia and thrombocytopenia account for less than 10% of all side effects. Laboratory tests often detect abnormalities in levels of aminotransferase and alkaline phosphatase. Elevated levels of histamine are a frequent side effect when using polypeptide-like compounds [7]. Echinocandins show embryo toxicity; therefore, these drugs should be avoided during pregnancy [64].

## 4. Resistance to Echinocandins

The occurrence of strains resistant to echinocandins was observed for the first time in 2005. Mutations in the *FKS* genes of resistant *C. albicans* (*FKS1*) and *C. glabrata* (*FKS2*), proven to decrease their sensitivity to caspofungin, have been detected [66]. The occurrence of resistance among *Candida* spp. varies depending on the species, the region of occurrence of infections, as well as the patient’s origin [67]. It is worth noting that among the strains of the *Candida* a low incidence of resistance is observed. According to Castanheira et al., resistance among *C. albicans* species is at the level of 3% [68]. In the case of *C. glabrata* isolates, which may show cross-resistance to azoles [67], an increase in resistance from 4.9% to 12.3% was demonstrated during studies conducted in the years 2001–2010 [69]. There have also been some cases of *C. krusei* strains resistant to echinocandins [70,71], and it is estimated that resistance in this species is about 2% [72]. The biggest decrease in susceptibility among fungi of the *Candida* is observed for *C. parapsilosis* and *C. guilliermondii* species [73]. However, infection by *C. auris* is now a serious problem. In 2016, the United States Centers for Disease Control and Prevention (CDC) issued a warning about the appearance of a multidrug resistant strain of *Candida* [74]. This pathogen was first isolated in 2009 from the ear canal during ear infection [75]. *C. auris* is the cause of nosocomial infections in many countries [76,77,78,79] and is associated with very high mortality [80]. The main problem is the high resistance to all drugs used in antifungal therapy. In the majority of studies, the MIC value for fluconazole exceeds 32 mg/L, and ≥ 2 mg/L for amphotericin B [27,80,81]. A recent study by Chowdhary et al. on 350 isolates showed that 90% of isolates were resistant to fluconazole, only 2% to echinocandins and 8% to amphotericin B [82]. However, echinocandins are used to treat *C. auris* infections after previous sensitivity tests [27,79]. In addition, an important problem is the incorrect identification of this species, which is often confused with *C. haemulonii*, *C. duobushaemulonii*, *C. sake*, *C. catenulata*, *C. famata* or even *C. parapsilosis* when using different methods of identification (Vitek 2 YST, 20C API, MicroScan, etc.) [83].

The main factor responsible for the emergence of resistance among *Candida* spp. seems to be prolonged or repeated exposure to echinocandins. The research confirms the relationship between the occurrence of mutations in the “hot spots” of the *FKS* gene and the exposure to echinocandins [84,85,86]. The use of echinocandins in antifungal treatment aimed at preventing infections may contribute to the development of resistance. Research carried out by Bizerra, as well as Ruggero and their associates, showed that small doses of the drug used in the prophylaxis were associated with the occurrence of echinocandin resistance among *C. glabrata* and *C. albicans* isolates [87,88].

*Candida* yeast’s biofilm structure is composed mainly of β-(1,3)-D-glucan, which limits the regular penetration of antibiotics into the cell. Too-low drug penetration can lead to strong selection pressure and the creation of resistant strains [89]. According to studies by Perlin et al., 40% of patients after multiple gastrointestinal surgery or pancreatitis developed resistant strains [90]. Genotyping results confirm that most patient infections are associated with commensal microorganisms of the digestive system [3].

### Reasons for the Occurrence of Echinocandin Resistance

One of the mechanisms of resistance is modification, resulting in a reduction of the effect of the antibiotic [91]. The occurrence of point mutations in specific regions of the *FKS* genes encoding the catalytic subunit, resulting in reduced sensitivity or the formation of echinocandin resistance, has been proven (Figure 2c) [2]. There are three genes coding the catalytic subunit of glucan synthase: *FKS1*, *FKS2* and *FKS3*. Shields et al. have shown the presence of mutations in the *FKS1* gene regions in all *Candida* species, and in the *FKS2* gene region of *C. glabrata* [86]. Mutations affecting susceptibility are found in two highly conserved regions, referred to as “hot spot” regions. Most often, amino acid substitutions occur at amino acid positions 641 to 649 and 1345 to 1365 with the FKS1 protein [28]. Mutations in the Ser 645 and Phe 641 positions account for 80% of all mutations detected in *C. albicans*, and are associated with the strongest phenotype [92,93]. In the case of *C. glabrata*, mutations occur twice as frequently in the FKS1 protein and they mostly result in Ser 629 and Ser 663, as well as Phe 659 as the result of mutations in FKS2 protein region. There are also missense mutations in both genes, which could lead to strong resistance among *C. glabrata* strains [28,69,93]. Mutations in the "hot spot" regions induce an increase in the MIC value by 10–100 times, and a reduction in the sensitivity of glucan synthase to echinocandins [92]. Mutations reduce the catalytic efficiency of glucan biosynthesis, resulting in changes in cell wall composition and cell morphology. In studies by Ben-Ami R. et al., *C. albicans* strains with a homozygous *FKS1* “hot spot” mutation were shown to have thicker cell walls, containing more chitin. In addition, these mutants show a reduced growth rate [94]. Mapping mutations within the “hot spot” on the topology map of the *FKS1* gene showed that amino acid substitutions occur near the surface of the extracellular membrane. This location may indicate enzyme interactions with echinocandins that would not have to enter the cell [92]. Less resistant phenotypes are observed when mutations occur near the C-terminus of the “hot spot” region [8]. Naturally occurring polymorphisms in the Pro 649 position in *C. parapsilosis*, as well as Met 633 and Ala 634 mutations, are responsible for high MIC values relative to other *Candida* spp. [3]. The presence of resistant strains is also observed in *Aspergillus* spp. Initial studies were based on the formation of mutants by a *FKS1* gene mutation, that results in a substitution at the amino acid position 678 (conversion of serine to tyrosine or proline). The obtained mutants were characterized by increased MEC values for three echinocandins [95]. However, in subsequent years, the occurrence of caspofungin resistance was observed among clinical isolates as well [28].

Increased gene expression of multidrug transporters is a common mechanism of azole resistance. However, it has been demonstrated that the gene transcription level of multidrug transporters is not related to the echinocandin resistance mechanisms [91]. Pfaller et al. carried out studies on azole resistant *C. albicans* strains, which were characterized by overexpression of genes encoding multidrug transporters. In vitro, these strains were shown to be highly sensitive to echinocandins [96,97]. This suggests the lack of clinical impact of multidrug transporters on the mechanism of resistance. However, according to studies, the overexpression of *CDR1*, *CDR2* and *MDR1* (encoding the transmembrane transporters occurs in azole resistant *C. albicans* strains) genes among *C. albicans* and *S. cerevisiae* strains indicates small changes in sensitivity to echinocandins, observed on solid media. Interestingly, these relationships were not observed during cultivation in liquid medium [98].

Another potential mechanism of echinocandin resistance is the initiation of cell response to stress. It is well known that fungi are not able to survive without a cell wall, thus maintaining the integrity of the cell wall is essential for the cell to survive [8]. Biosynthesis and repair of the cell wall is characterized by high dynamics, which is regulated by the cell cycle, their morphogenesis, and also stress factors [99]. The decrease in β-(1,3)-D-glucan synthesis induces cellular stress, due to the lack of continuity of the cell wall. In response to stress, adaptive mechanisms are activated to protect the cell from environmental stress [3]. Signals are then transmitted in the cell and reach the RHO1 protein subunit, which regulates the activity of glucan synthase as well as coordinating the action of PKC protein. PKC is responsible for periodic reconstruction of the cell wall depending on the cell cycle. Research indicates the role of PKC in maintaining the integrity of the cell wall through the synthesis of a compensatory cell wall, made of chitin (Figure 2d) and mannan [91]. Increased chitin synthesis in response to destruction of the cell wall can also be coordinated by MAPK and calcineurin. MAPK participates in responses to oxidative and osmotic changes. The calcineurin pathway is activated with calcium. Activated calcineurin dephosphorylates CRZ1 protein, a transcription factor that moves to the nucleus and induces gene expression [28]. For most *Candida* species, activation of the *CHS2* and *CHS8* genes allows cell survival in the presence of growth-inhibiting concentrations of echinocandins [3]. It is likely that the increase in the level of chitin in the cell wall may be related to the previously described "paradoxical" growth of strains in the presence of echinocandins at a concentration well above the determined doses [3,91]. It is possible that adaptive mechanisms stabilize the cell during its presence in the drug environment and allow the cell to minimize the effects of the drug by generating mutations in the “hot spot” *FKS* gene [3].

*C. auris*’ multidrug resistance mechanism is not yet well understood. Sequencing the genome of this strain showed the presence of a large number of genes encoding ATP binding cassette (ABC) family membrane carriers and major facilitator superfamily (MFS), which are important in azole resistance [27]. Furthermore, kinase-coding genes, such as the genes that encode *HOG1* or protein kinase A, have been observed to contribute to echinocandin tolerance in *C. albicans* [100]. A recent multicenter study indicated that the mechanism of *C. auris* resistance may be mutations in genes that are molecular targets for antimycotics. In 77% (34/44) of fluconazole-resistant strains, mutations Y132 and K143 were found in the *ERG11* gene encoding lanosterol 14-α demethylase. Those strains showing lower MIC values for fluconazole (1–2 mg/L) did not have those mutations. After exposure to fluconazole, no increase in the expression level of the *ERG11* gene was observed. The new S639F mutation in the *FKS1* gene in “hot spot” 1 was correlated with the lack of sensitivity to echinocandins [82].

## 5. Next-Generation Echinocandins

**Figure 3:**
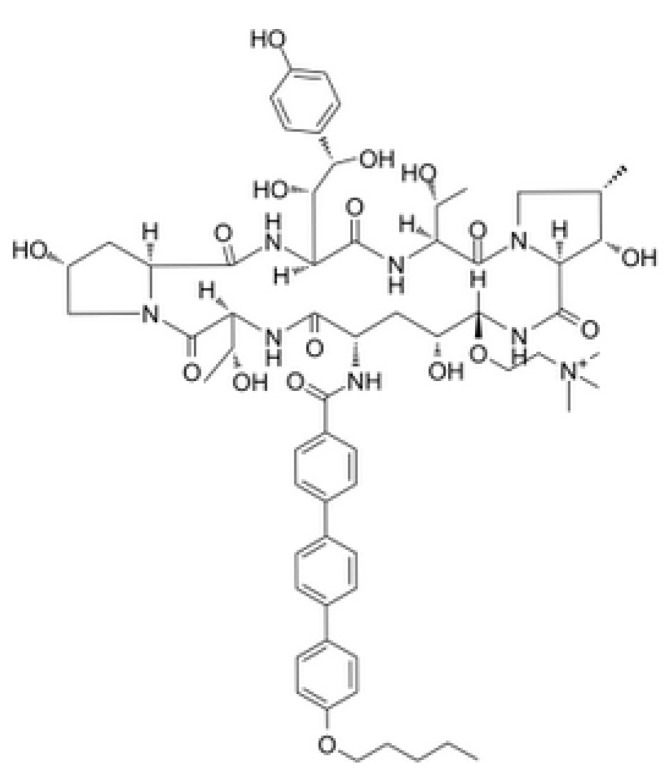
Chemical structure of rezafungin.

Rezafungin, previously called CD101 (Cidara Therapeutics), is a currently developing novel molecule in the echinocandin class. It is a structural analogue of anidulafungin, consisting of cyclic hexapeptide with a lipophilic tail and choline moiety at the C5 ornithine position (Figure 3) [101]. The changes in the structure influence increased chemical stability in plasma, aqueous solution and also elevated temperature [101]. Moreover, this echinocandin has a long half-life [102]. Rezafungin displays in vitro potency and a spectrum of activity the same as that in already used echinocandins. The pharmacokinetic profile enables once-weekly intravenous formulation, for the treatment and prevention of systemic fungal infections [103]. 

Rezafungin susceptibility testing of wild-type and antifungals-resistant fungal isolates was performed, with EUCAST and CLSI recommendations [102,104,105,106,107]. The activity potential of rezafungin against *Aspergillus* spp. was comparable to anidulafungin, but was four-fold more active than caspofungin [102]. A minimum effective concentration of rezafungin MEC_90_ ≤0.008–0.03 mg/L was reported against *A. fumigatus*, *A. terreus*, *A. niger* and *A. flavus*. The same research demonstrated that the activity of rezafungin against the most frequent *Candida* spp. is comparable to other members of the echinocandin class. Data based on *Candida*, *Aspergillus* and *C. neoformans* isolates collected worldwide in 2014 and 2015 confirm preliminary observations about rezafungin susceptibility [39,104,105]. According to the collected data, it appears that the upper limit of WT MIC distributions for *Candida* spp. was ≤0.12 mg/L, but for *C. parapsilosis* and *C. orthopsilosis* was ≤4 mg/L, and the MEC was ≤0.03 mg/L for *Aspergillus* spp. [39,105]. Rezafungin also exhibited activity against biofilm, through reductions in biofilm thicknesses in mature and early phases and also through inhibition of the formation of biofilm during adhesion [108]. Development of rezafungin resistance was investigated using spontaneous resistance and the serial exposition to rezafungin, anidulafungin and caspofungin of five isolates of *C. albicans*, *C. glabrata*, *C. krusei* and *C. parapsilosis* [109]. The median frequency of spontaneous and one-step mutations contributing to reduced rezafungin sensitivity was 1.35 × 10^−8^ to 3.86 × 10^−9^. Moreover, cross-resistance to anidulafungin and caspofungin has been observed among the mutants, which may suggest the absence of unique mutations for rezafungin. This new-generation echinocandin also has a potential use against multi-resistant *C. auris* isolates. The rezafungin susceptibility of most isolates had a modal MIC value of 0.25 mg/L. Moreover, some activity of this echinocandin was reported among *C. auris* isolates with a higher MIC than modal MIC value, but rezafungin was not active against isolates with the S639P *FKS1* mutation [110]. Rezafungin is also active against *C. auris* isolates resistant to fluconazole and amphotericin B [111]. 

Currently, the third phase of clinical development of rezafungin is in progress. Phase 3 is a randomized, double-blind, multicenter clinical study on the efficacy and safety of rezafungin for injection, compared with available intravenous caspofungin, followed by an oral reduction in the dose of fluconazole, in the treatment of candidemia and invasive candidiasis. (ClinicalTrials.gov registration no. NCT03667690). Greater stability of the drug is expected to improve the effectiveness of the drug, especially at the beginning of treatment, where the pathogen density is high [102]. Prolonged activity increases the rate of pathogen killing, reducing spontaneous mutations and eliminating pre-existing drug resistant subpopulations. On the other hand, less frequent dosing will contribute to reducing the cost of treatment. Rezafungin has the potential as a new-generation antifungal agent, with novel properties that can face the challenges in the treatment and prevention of invasive fungal infections.

It is worth noting that β-(1,3)-D-glucan synthase is the molecular target not only for echinocandins. The example is SCY-078 (ibrexafungerp), belonging to triterpenoid class. It is a semisynthetic derivative of the naturally occurring enfumafungin [112]. A phase 3, multicenter, randomized, double-blind and placebo-controlled study, to evaluate the efficacy and safety of oral ibrexafungerp compared to placebo in subjects with recurrent vulvovaginal candidiasis, is in progress (ClinicalTrials.gov no. NCT04029116). In vitro, SCY-078 has shown a broad spectrum of activity against the clinical isolates of *Candida* spp. and *Aspergillus* spp. [113]. Importantly, this compound also demonstrates activity against the majority of *Candida* isolates harboring the *FKS* gene mutations resistant to echinocandins [114,115], as well as against azole resistant isolates [116]. This drug also showed activity against a multidrug resistant strain of *C. auris* [117].

## 6. Conclusions

Antibiotics from the echinocandin group are highly effective and are less harmful compared to other drugs. It is worth noting that the treatment of invasive candidiasis by micafungin [118] and caspofungin [119] is economically advantageous compared to the use of amphotericin B. Furthermore, the additional advantage of those drugs is the possibility of their use in patients with impaired renal function. However, the use of anidulafungin may be more cost-effective in the treatment of invasive candidiasis compared to fluconazole [120].

Despite the fact that the number of reports on the occurrence of echinocandin resistant strains among *Candida* spp. is increasing, only a few clinical failures were reported. Recently, there has been an increase in the MIC value for some strains, which may be related to the patient’s long-term exposure to echinocandin drugs. Therefore, it is important to distinguish the adaptive mechanisms of fungi that increase MIC in vitro from mechanisms that affect clinical failures [8]. Understanding the clinical and molecular factors that are responsible for the emergence of resistance among strains is key to developing better therapeutic tools.

## Figures and Tables

**Table 1:** Breakpoints of echinocandins established by EUCAST and CLSI.

Antifungal Agent	Standard	*C. albicans*		*C. glabrata*		*C. krusei*		*C. parapsilosis*		*C. tropicalis*	
		S≤	R>	S≤	R>	S≤	R>	S≤	R>	S≤	R>
AND	EUCAST	0.03	0.03	0.06	0.06	0.06	0.06	4	4	0.06	0.06
	CLSI	0.25	0.5	0.12	0.25	0.25	0.5	2	4	0.25	0.5
CAS	EUCAST	N	N	N	N	N	N	N	N	N	N
	CLSI	0.25	0.5	0.125	0.25	0.25	0.5	2	4	0.25	0.5
MCF	EUCAST	0.016	0.016	0.03	0.03	IE	IE	2	2	IE	IE
	CLSI	0.25	0.5	0.06	0.12	0.25	0.5	2	4	0.25	0.5

IE—Insufficient evidence; N—until caspofungin breakpoints have been established, susceptibility of this echinocandin should be considered based on susceptibility of the remaining two echinocandins. So, if the isolate is susceptible to anidulafungin as well as micafungin, it should be considered susceptible to caspofungin.

**Table 2:** Spectrum of activity against common fungi.

	Antifungal Agent			
Organism	AND	CSP	MCF	Reference
*Candida albicans*	+	+	+	[38,39]
*Candida glabrata*	+	+	+	[39]
*Candida parapsilosis*	+	+	+	[39]
*Candida tropicalis*	+	+	+	[39]
*Candida krusei*	+	+	+	[39]
*Candida lusitaniae*	+	+	+	[38]
*Aspergillus fumigatus*	+	+	+	[40]
*Aspergillus flavus*	+	+	+	[40]
*Aspergillus niger*	+	+	+	[40]
*Aspergillus terreus*	+	+	+	[40]
*Acremonium*	-	-	-	[35,41]
*Alternaria*	-	-	-	[35]
*Blastomyces* spp.	+/−	+/−	+/−	[34]
*Coccidioides* spp.	+/−	+/−	+/−	[36]
*Cryptococcus neoformans*	-	-	-	[32,33]
*Curvularia*	+	+	+	[42]
*Fusarium* spp.	-	-	-	[40]
*Histoplasma* spp.	+/−	+/−	+/−	[17]
*Mucorales*	-	-	-	[43]
*Rizpous*	-	-	-	[43]
*Scedosporium* spp.	-	-	-	[17]
*Trichoderma*	+	+	+	[44]
*Trichosporon*	-	-	-	[17,45]

+/− The agent has variable activity against the organism.
